# A Junior Course of Practical Zoology

**Published:** 1899-12

**Authors:** 


**A Junior Course of Practical Zoology.** By the late A. MILNES MARSHALL, M.D., F.R.S., and the late C. HERBERT HURST, Ph.D. Fifth Edition. Revised by F. W. GAMBLE. Pp. xxxv., 486. London: Smith, Elder, & Co. 1899.

Owing to the early death of Dr. Hurst, following as it did so comparatively shortly after that of Dr. Milnes Marshall, the task of bringing out the fifth edition of this well-known and valuable work has fallen to Mr. Gamble. No new figures have been added, and, as is mentioned in the preface, the alterations in this latest edition are mainly in the introductory chapters on technique, which have been recast. A word of special praise should be given to the index, which is admirable in its completeness.

